# Marine Peptides: Potential Basic Structures for the Development of Hybrid Compounds as Multitarget Therapeutics for the Treatment of Multifactorial Diseases

**DOI:** 10.3390/ijms252312601

**Published:** 2024-11-23

**Authors:** Marta Bauer, Magdalena Glowacka, Wojciech Kamysz, Patrycja Kleczkowska

**Affiliations:** 1 Department of Analytical Chemistry, Faculty of Pharmacy, Medical University of Gdansk, 80-416 Gdansk, Poland; 2 Institute of Psychology and Human Sciences, WSEI Academy, 20-209 Lublin, Poland; magdalena.glowacka@wsei.pl; 3 Department of Inorganic Chemistry, Faculty of Pharmacy, Medical University of Gdansk, 80-416 Gdansk, Poland; wojciech.kamysz@gumed.edu.pl; 4 Maria Sklodowska-Curie Medical Academy in Warsaw, 03-411 Warsaw, Poland

**Keywords:** marine peptides, hybrid, efficacy, tolerability, safety profile

## Abstract

Marine-derived peptides display potent antihypertensive, antioxidant, analgesic and antimicrobial biological effects. Some of them have also been found to have anticancer activity via various mechanisms differing from those of continental organisms. This diversity of properties—together with the peptides’ efficacy, which has been confirmed in several in vitro and in vivo studies—make these compounds attractive as functional ingredients in pharmacy, especially in regard to multitarget drugs known as hybrids. Given the possibilities offered by chimeric structures, it is expected that a hybridization strategy based on a marine-derived compound could result in a long-awaited success in the development of new effective compounds to combat a range of complex diseases. However, despite the fact that the biological activity of such new hybrids may exceed that of their parent compounds, there is still an urgent need to carefully determine their potential off-targets and thus possible clinically important side effects. Given the above, the aim of this paper is to provide information on compounds of marine origin with peptide structures and to verify the occurrence and usage of hybrid compounds built from these structures. Furthermore, the authors believe that information presented here will serve to increase public awareness of the new opportunities arising from the combination of hybridization strategies with marine molecules with known structures and biological properties, thereby accelerating the development of effective drug candidates.

## 1. Introduction

Despite significant medical advances in the development and production of new drugs, nowadays, when multiple diseases are becoming increasingly common in society, as are multiple mechanisms of each disease, commonly used therapeutic agents have become sometimes ineffective or even fatal. In many cases, it turns out that a single drug does not have the necessary effect to combat the cause of the disease, and the use of additional pharmacological substances leads to either inhibitory or reinforcing interactions.

This so-called polytherapy poses many problems for patients and prescribers. However, it is definitely more difficult for the former, as there is an increasing lack of cooperation between doctors of different specialties treating a given patient. On the one hand, polytherapy is thought to enhance treatment efficacy, owing to possible drug interactions with multiple molecular targets, and thus, achieving an improved response through its multidirectional effects. On the other hand, simultaneous use of single or multiple drugs can lead to fatal side effects that, as already mentioned, are related to the pharmacokinetics and/or pharmacodynamics of drug–drug interactions. The use of two active ingredients in a single tablet (so-called ‘multi-substance medicine’) attempts to mitigate possible interactions but does not solve the problem of side effects. Although refining a therapy by reducing the number of medications taken improves so-called ‘compliance’—the cooperation between a patient and doctor, which seems to be extremely important—especially in patients who do not fully adhere to their doctor’s recommendations, the therapeutic efficacy of this type of medication ultimately remains questionable.

Hence, it seems important to develop a drug candidate with improved efficacy while maintaining the required safety profile. In this regard, hybrid structures, also known as designed multiple ligands (DMLs) or chimeras, appear to be ideal. The hybridization (conjunction) strategy is based on the design and development of a drug that combines two different biologically active molecules acting on different targets, so that the effect produced by each molecule also becomes combined (e.g., additive or synergistic effect) [1,2,3,4].

Recently, the hybrid approach has been frequently applied to natural compounds, including endogenous bioactive compounds, such as neurotransmitters and/or neuromodulators (e.g., endomorphins, substance P) [5,6,7], plant-derived compounds (e.g., terpenoids, β-triketone, phenols) [8,9] or even molecules originating from animals (e.g., botulinum toxin, bleomycin, insulin) [10]. In this context, marine-derived compounds, in particular, have attracted much attention, as they are characterized by a wealth of beneficial effects, such as antimicrobial, cytotoxic, anti-inflammatory, analgesic, antiparasitic and multiple enzyme inhibitory activities [11,12,13,14,15,16]. Their combination with other biologically active structures in a single entity could produce a potent drug candidate useful for the treatment of a specific disorder that is also characterized by relatively well-tolerated pharmacokinetic and pharmacodynamic profiles.

## 2. Hybridization Strategy

The adverse effects caused by taking a drug are essential considerations for any pharmacotherapy. They are not only the result of improper drug use but also of genetic polymorphisms that significantly alter the therapeutic effect. Adverse drug reactions can also occur with correctly administered remedies, especially when several drugs are taken at short intervals or, sometimes even, simultaneously.

Potential solutions to the above problem could therefore be sought in hybrid structures, increasingly proposed as effective prescriptions for combating multiple co-occurring diseases and disorders. Importantly, they have also been found to be highly effective in the treatment of a single disease, even if the disease has a multifactorial etiology (e.g., schizophrenia or multiple sclerosis (MS), which is additionally characterized by psychiatric symptoms, pain or urinary tract infections).

**Figure 1:**
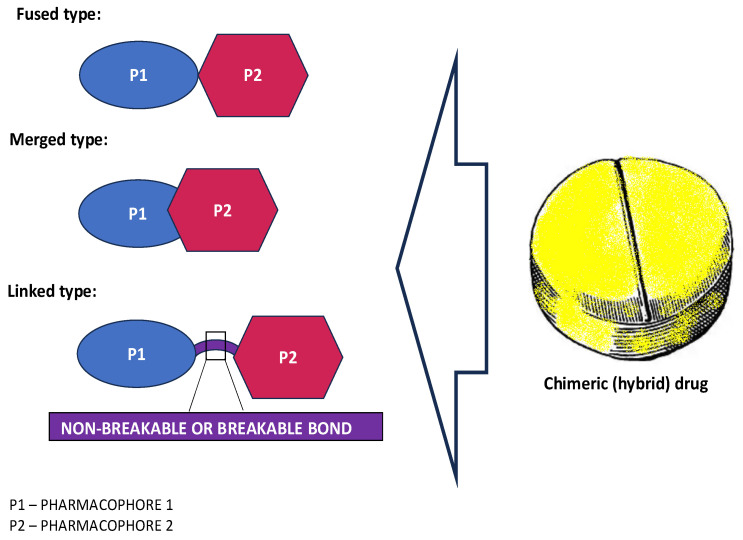
Types of hybrid molecules according to that location of their structural pharmacophores (P) in the scaffold. Fused hybrids mean that two biologically distinct pharmacophores are directly linked by the functional group of each fragment. Merged hybrids have components that overlap each other (the merged pharmacophores usually have similar elements/sequences), resulting in a smaller and simpler molecule, while linked hybrids are molecules that are bridged by a spacer that can be enzymatically stable (e.g., alkyl chain, aryl fragments) or can be cleaved, such as ester and disulfide bonds.

The combination of the two structures in a single molecule is intended not only to minimize undesirable effects that could result from taking the given medication in a mixed form, but also to enhance the positive effect of the medication required in individual cases. Of course, it is extremely important here to distinguish between hybrid and multitarget compounds, whose numbers are constantly increasing in the market. Structurally, multitarget compounds, except bivalent hybrid molecules, do not have specific building blocks that correspond to original drugs or other known ligands. Instead, they are characterized by recognized and privileged small fragments/regions that are capable of targeting a molecule to different systems (e.g., heterocyclic cores, aliphatic residues). In contrast, hybrid compounds, as just mentioned, represent combinations of two or more bioactive structures from a different class or their pharmacophoric subunits in a single molecule, representing the desired properties of original drugs (Figure 1). Although there are structural features that can distinguish the two types of molecules, the overall distinction is very fluid, as hybrid compounds are also multitargeted in terms of their ability to affect multiple biological targets and, thus, generate a broad spectrum of biological activities.

Regardless of the type of hybridization (i.e., fused, merged or linked), these novel compounds have been shown to offer certain advantages, especially compared to those combination therapy drugs. These advantages include enhanced molecule bioactivity, often owing to synergistic interactions between their individual component activities [3,16,17,18], leading to improved efficacy. Of note, such a synergistic effect can meet the treatment needs of complex diseases. In addition, a better pharmacokinetic profile is achieved and, in the case of pharmacodynamics, the risk of side effects, side effect exacerbation as well as possible drug–drug interactions is lower than with a drug cocktail [19]. Furthermore, hybridization can confer supplementary physicochemical properties absent in a single structural unit. Nevertheless, it should be considered that such a new compound is also a known molecule that has the potential to alter the pharmacological spectrum of the chimera (i.e., unexpected new functionalities might be seen) [20,21]. Therefore, molecular targets that were not recognized and not associated with a structural motif of a single pharmacophore could then be influenced by the drug [3,21,22,23]. Unfortunately, this also means that off-target adverse effects need to be kept in mind.

## 3. Marine Peptides—Characteristics and Biological Activity of Selected Compounds

Peptides are a class of compounds that, in addition to some advantages, have a number of serious limitations, such as conformational instability, a short half-life and inadequate penetration across the blood–brain barrier (BBB) [24,25,26,27]. In addition, most of these molecules are characterized by rapid hepatic and renal clearance and inadequate passive transport across cell membranes. All these features contribute to the reduced absorption and distribution of the drug in the body. In addition, the absence of resistance of peptides to degradation by gastrointestinal proteolytic enzymes (e.g., carboxypeptidases or aminopeptidases) should be mentioned, which, in practice, might make oral administration of such a drug to the patient impossible and necessitate intravenous administration [26,28].

Marine-derived peptides have several advantages over those isolated from non-marine (continental) animals or plants and offer, therefore, an invaluable source of molecules on the basis of which an effective drug candidate can be designed. For instance, they are characterized by a unique amino acid composition that makes them resistant to proteolytic enzymes [29], so they have a better chance of reaching their target site in an intact form. They form both linear and cyclic structures [30,31]. They usually consist of a great number of prolines responsible for the compound’s stability [32,33,34]. In addition, some of them, in particular peptides with antimicrobial properties, are rich in lysine and/or arginine, both of which give the peptide a positive charge [35,36,37]. Marine-derived peptides are relatively small in relation to their molecular weight, making them better at overcoming physiological barriers. One of their other advantages over other peptide sources is the presence of *D*-amino acids [38], which provide additional enzymatic stability to the structure and make the peptide less immunogenic as compared to that of *L*-peptides [39,40]. Furthermore, given the diverse living conditions of marine organisms (e.g., high-pressure, hypersaline or low oxygen environments, etc.), which provide them with unexpected survival opportunities to grow, reproduce and defend themselves, their peptides have been shown to exert therapeutic effects via a very unique and different mechanism of action than that of other animal-derived peptides [41,42]. It should be noted that marine organisms such as marine cyanobacteria, marine fungi, sponges or algae also contain depsipeptides that are either cyclic or linear. These form a new subclass of peptide compounds consisting of amino acids with at least one ester bond replacing a peptide bond. This could be the cause of their different pharmacological activities as compared to that of unmodified peptides [43,44,45].

There are a large number of potent peptide compounds that occur naturally in marine organisms. However, only a few will be presented here to show how great the therapeutic potential of marine life is. It should be noted that marine organisms are also known to be a great source of peptides with various biological activities when producing protein hydrolysates; this includes macroalgal, fish and shellfish processing waste byproducts. A proper example in this context is angiotensin I-converting enzyme (ACE)-inhibiting peptides (i.e., Leu-Lys-Leu, Lys-Val-Leu-Ala-Gly-Met, Leu-Lys-Val-Gly-Val-Lys-Gln-Tyr) from a sardine muscle hydrolysate [46,47] or the nine-amino acid peptide Leu-Gly-Leu-Asn-Gal-Asp-Asp-Val-Asn from conger eel muscle hydrolysate, which exhibits antioxidant properties [48]. Of course, there are also reports on the isolation of other peptides from a variety of other marine-derived hydrolysates, such as peptides that inhibit HIV-1 protease and compounds with anti-anemic activity, etc. Hence, protein hydrolysates should be thoroughly investigated as a new, additional source of potentially biologically active compounds.

### 3.1. Fish Peptides

Fish appear to be a readily accessible source of biologically active compounds among other marine organisms. In fact, fish protein hydrolysates (FPH) have been reported to consist of a variety of components, including peptides, specific bioactive lipids, fatty acids and trace minerals. These, in turn, can provide valuable elements with potential nutritional or pharmaceutical applications.

Tuna has long been known as a high-quality source of proteins and peptides. For example, tuna was the source of the first ever reported marine ACE-inhibitory peptide with the amino acid sequence Pro-Thr-His-Ile-Lys-Trp-Gly-Asp, showing efficacy at an IC_50_ of 2 μM [49]. Recently, Guo and colleagues characterized 54 tuna-derived peptides of different lengths and sequences that exhibited antioxidant activities [50] (Table 1). They suggested, similar to Kim et al. [51], that the molecular weight of a peptide is an important factor influencing its antioxidant properties. Hence, the lower the molecular weight, the more effective the interaction between the peptide and free radicals. Other tuna peptides, such as Leu-Pro-His-Val-Leu-Thr-Pro-Glu-Ala-Gly-Ala-Thr and Pro-Thr-Ala-Glu-Gly-Gly-Val-Tyr-Met-Val-Thr, have also been reported to have an inhibitory effect on the proliferation of MCF-7 human breast cancer cells, with a corresponding IC_50_ values of 8.1 and 8.8 μM, respectively [52]. This antiproliferative activity was confirmed by further studies by Zhao et al. [53], but in this case, a total tuna protein hydrolysate (TPA) consisting of 109 peptides, such as Gly-Met-Asp-Val-Ile-Asn-Met, Val-Met-Ala-Pro-Gly-Ala-Gly-Val-Tyr and Glu-Val-Met-Ala-Pro-Gly-Ala-Gly-Val-Tyr was investigated. Moreover, it was shown that a combination of TPA with 5-fluorouracil (5-FU), when administered subcutaneously to male BALB/c mice bearing sarcoma S180 cells, resulted in the significant inhibition of tumor growth as compared to 5-FU monotherapy. In addition, TPA and 5-FU were found to reduce intestinal mucosal injury induced by 5-FU.

Tuna dark muscle hydrolysates are also rich in peptides that have an ACE-inhibiting effect. Some of the most potent compounds were isolated and identified by Lee and colleagues [54], as well as by Suo et al. [55]. For instance, a Gly-Asp-Leu-Gly-Lys-Thr-Thr-Thr-Val-Ser-Asn-Trp-Ser-Pro-Pro-Lys-Try-Lys-Asp-Thr-Pro peptide with IC_50_ 11.28 μm was discovered [54]. Significantly shorter compounds, such as Ile-Cys-Tyr and Leu-Ser-Phe-Arg, were also found to be active (Table 1). Here, too, the molecular weight of the compound proved to be decisive for its activity. Furthermore, as presented by the sequence, it was demonstrated that the type of amino acids, either *N*-terminal or *C*-terminal, could have an influence on the overall inhibitory activity against ACE. Indeed, branched aliphatic amino acids at the *N*-terminus could improve the ACE-inhibitory effect of the peptides, while *C*-terminal amino acid residues containing a positive charge, such as Arg, could be conducive to elevated inhibition against ACE.

It is worth noting that similar antihypertensive effects were demonstrated for peptides from shark meat hydrolysates (Table 1) [56]. However, shark, in particular its cartilage, is well-known for its strong antiangiogenic and antitumor activities [57]. In this context, it was discovered that troponin I (peptide Glu94-Leu123; pTnI), a molecule responsible for inhibition of the actomyosin ATPase during muscle contraction, inhibited pancreatic cancer metastases in an in vivo liver metastasis model [58]. Other attractive features of shark-derived peptides include hepatoprotective and antidiabetic activities. As demonstrated by Huang et al. [59], the shark liver peptide S-8300 (Table 1) effectively reduced alanine transaminase (ALT) and aspartate transferase (AST) levels while elevating superoxide dismutase (SOD) and glutathione (GSH) in a mouse model of CCl4-induced hepatotoxicity. The same authors investigated the behavior of S-8300 in alloxan diabetic mice and found its activity to be similar to that reported for insulin [60].

Many fish species have been known to be the source of antimicrobial peptides. This is the case of the Red Sea Moses sole (*Pardachirus marmoratu*) containing pardaxin, a 33-amino acid linear polypeptide (Table 1), which has time- and dose-dependent antitumor activity, as shown by Huang et al. [61] and others [62].

Potent antimicrobial activity against a variety of microorganisms was reported for piscidins, histidine-enriched peptides identified in teleost fish taxa [63,64], and for hepcidins, which, in contrast, are cysteine-rich peptides. Also, both types of compounds exhibited a broad range of activities not only associated with antifungal, antiparasitic and antiviral properties [65,66,67,68,69] but also closely related to the inhibition of cancer cells [70,71,72,73].

There are many peptides that either occur naturally in fish of different species or are produced by the enzymatic hydrolysis and microbial fermentation of their proteins. Their bioactivity depends largely on physicochemical conditions used for their isolation. Therefore, it is quite difficult and rather impossible to indicate precisely the total number of possible bioactive fish-derived peptides. Nevertheless, Table 1 presents some examples of peptide sequences with beneficial therapeutic effects.

**Table 1:** Examples of biologically active marine peptides and their activities.

Peptide Source	Peptide Sequence (One-Letter Amino Acid Code)	Displayed Activity	Ref.
Tuna	AEPAPAPAPAPEPAPAPA, GEPGPAG, LPGGGPVL, AAAPAPAPAPAPA, AGLYPGA	antioxidative	[50,51]
	LPHVLTPEAGAT and PTAEGGVYMVT	anticancer	[52]
	ICY, LSFR, IYSP	antihypertensive (ACE-inhibitory) and antioxidative	[55]
	GILTLK	antimicrobial	[74]
	WPEAAELMMEVDP	antioxidative	[75]
	GDLGKTTTVSNWSPPKYKDTP	antihypertensive	[54]
Mackerel	LDIQKEV, TAAIVNTA	antioxidative	[76]
Shark	CF, EY, MF, FE	antihypertensive (ACE-inhibitory)	[56]
	MLVGPIGAAKVVYEQ-XX X—unknown amino acid residues not defined by the authors	hepatoprotective, immunomodulatory, antidiabetic, antioxidative	[60]
Sole	GFFALIPKIISSPLFKTLLSAVGSALSSSGGQE (called pardaxin)	antimicrobial, antitumor, increase in dopamine release	[61,77,78,79]
	MIFPGAGGPEL	antihypertensive	[80]
Hagfish	GWFKKAWRKVKNAGRRVLKGVGIHYGVGLI	antimicrobial (including antifungal activity)	[81,82]
Cod	TGGGNV, TCSP	antioxidative, ACE-inhibitory	[83]
Herring	PPVEEP, GPAGDPA, GADPEDVIVS	antidiabetic	[84]
Salmon	WA, WM, VW, MW, IW, LW, FL	ACE-inhibitory	[85]
	GPAE	antidiabetic	[86]
Sardine	LKVGGKGY, LY, YL, GRP, RFH, GWAP	ACE-inhibitory	[87]

### 3.2. Marine Snail Peptides

Marine snails are a well-known source of several bioactive compounds, such as serotonin and vasopressin/oxytocin-related peptides [88,89]. However, some of the most important biologically active components are marine peptide toxins, including conotoxins, conopressins and conantokins; these usually occur in *Conus* species (i.e., fish-hunting, worm-hunting, and mollusk-hunting species) [90]. Therefore, much work has been expended to isolate and examine the pharmacological profile of these compounds.

The first marine-derived drug, Ziconotide (brand name Prialt^®^, the Primary Alternative’ to morphine, also known as SNX-111) approved by the Federal Food and Drug Administration (FDA) in December 2004, was a 25-amino acid, cyclic ω-conopeptide (Cys-Lys-Gly-Lys-Gly-Ala-Lys-Cys-Ser-Arg-Leu-Met-Tyr-Asp-Cys-Cys-Thr-Gly-Ser-Cys-Arg-Ser-Gly-Lys-Cys-NH2) with three disulfide bonds that originates from tropical venomous marine cone snails (*Conus magus*) [91]. The peptide has unprecedented selectivity for a calcium channel subtype that had not previously been recognized (known as the *N*-type calcium channel and, later, as Cav 2.2). Consequently, this molecule turned out to have potential as an effective non-opioid analgesic for pain prevention, as it demonstrated considerable efficacy in both neuropathic and cancer-related pains [92]. In addition, intrathecal administration of ziconotide prevented mechanical and cold allodynia, as well as heat hyperalgesia in neuropathic rats [93]. Unfortunately, the drug showed some side effects at therapeutic doses. In fact, mild ataxia, urinary retention and auditory hallucinations, as well as dose-related psychosis, could be diagnosed.

Among other conopetides, a μ-conotoxin (geographutoxin II, GTXII) from the *Conus geographus* marine snail should also be noted. In fact, this peptide, composed of 22 amino acid residues that includes 3-hydroxyprolines (Arg-Asp-Cys-Cys-Thr-Hyp-Hyp-Arg-Lys-Cys-Lys-Asp-Arg-Arg-Cys-Lys-Hyp-Met-Lys-Cys-Cys-Ala-NH2), blocks voltage-sensitive Na channels in the cell membrane of skeletal muscles and autonomic nerves [94]. Of note, based on a study demonstrating that the peptide interacts competitively with saxitoxin in binding at neurotoxin receptor site 1 of the Na channel in a highly tissue-specific manner, GTXII was proposed to be able to discriminate between nerve and muscle Na channels [94,95]. Moreover, at doses ranging from 3 × 10^−9^ to 10^−7^ M, it inhibited twitch responses to direct stimulation in an isolated mouse diaphragm [96,97]. It was also suggested that GTXII at higher concentrations inhibited chemical transmitter release from some kinds of nerve cells [94].

In contrast, another snail peptide, although linear, was found to serve as an antagonist towards *N*-methyl-*D*-aspartate receptors (NMDARs). Conantokin-G (also referred to as CGX-1007) was effective in suppressing seizures in various animal models [98]. Also, based on its mechanism of action, it exerted antinociceptive effects at doses 10 times lower than those associated with motor impairment and 20 times lower than those associated with side effects in models of injury-invoked pain [99].

### 3.3. Algae and Macroalgae Peptides

Micro- and macroalgae peptides, also known as cryptides [100], are endogenous compounds that also offer health benefits. In fact, using various enzymes for the generation of such peptides from parent proteins and polypeptides, such as pepsin, trypsin or papain, resulted in a wide range of different bioactive structures with broad activity.

For instance, trypsin hydrolysis of proteins from *Porphyra haitanesis* was shown to generate anticancer peptides. A typical example is a peptide sequence of Val-Pro-Gly-Thr-Phe-Lys-Asn-Leu-Asp-Ser-Pro-Arg, which turned out to be a more potent drug candidate in an in vitro human hepatocellular carcinoma HepG2 model than was 5-fluorouracil at IC_50_ of 200.97 μg/mL [101].

Pepsin was also used in the enzymatic hydrolysis of protein extracts from the microalgae *Chlorella vulgaris* and *Spirulina platensis* [102]. This step led to several short peptides with ACE-inhibitory activity. In fact, the tetrapeptide Ile-Ala-Pro-Gly and tripeptide Phe-Ala-Leu were each found to be potent and long-lasting in vivo. Another effect of pepsin, but also trypsin and chymotrypsin and that is used in enzymatic hydrolysis and carried out with the microalgae *Isochrysis zhanjiangensis*, yielded a compound with antioxidant activity in an in vitro alcohol-induced injury model [103]. Chen et al. [103] revealed the compound’s sequence as Asn-Asp-Ala-Glu-Tyr-Gly-Ile-Cys-Gly-Phe and found that this decapeptide acted through direct interaction with glutathione and superoxide dismutase, as the level of these enzymes turned out to be elevated.

## 4. Marine Peptide-Based Hybrids—Are There Any?

The number of hybrid structures composed of marine-derived compounds is dramatically low, although—as mentioned above—marine-sourced molecules are recognized as an excellent basis for the discovery and development of new and highly bioactive compounds with a broad spectrum of activity. Interestingly, such chimeric structures have been shown to be natural components in the secretions of marine organisms, which might explain their unique biological activities and mechanisms of action, which sometimes significantly differ from those of compounds identified in continental organisms.

### Marine Peptide-Based Hybrids and Their Efficacy in Preclinical Studies

One of the first hybrid compounds consisting of marine peptides was a SNX-202 chimera described by Ramachandran in 1994 [104]. It consists of a truncated fragment of the naturally occurring ω-conopeptide SNX-111 (ziconotide), isolated from the venom of the marine snail *Conus magus*, and its modified analog, SNX-183, which corresponds to SVIB peptides from *Conus striatus* [104,105] (Table 2). Interestingly, this new peptide proved to be an antagonist of noradrenaline release, as it binds to neuronal *N*-type voltage-sensitive calcium channels (VSCC) with a 30-fold higher affinity than that of SNX-183 in competitive binding experiments on ex vivo rat brain synaptosomes [104]. Since the mechanism of action of conotoxins is mainly based on an interruption of activity of the three main ion channels (i.e., Na^+^, Ca^2+^ and K^+^) involved in pain transmission, among others, the hybridization strategy applied to these snail peptides was shown to produce not only analgesic effects without motor deficits in animals—as demonstrated by original compounds [106,107]—but also to treat other pathological conditions, depending on the structure of the chimera’s second pharmacophore.

Tombaccini and colleagues were engaged in a completely different research field with a bicyclic ω-conopeptide of 27 amino acids, which was identified as the GVI A toxin from *Conus geographus* and *Conus magus*. This time, the conotoxin was used to produce monoclonal antibodies against the toxin [108]. In this context, GVI A was covalently conjugated to bovine serum albumin (BSA) and the ex vivo studies carried out showed that it inhibited the binding of free conotoxin to rat brain synaptosomes.

**Figure 2:**
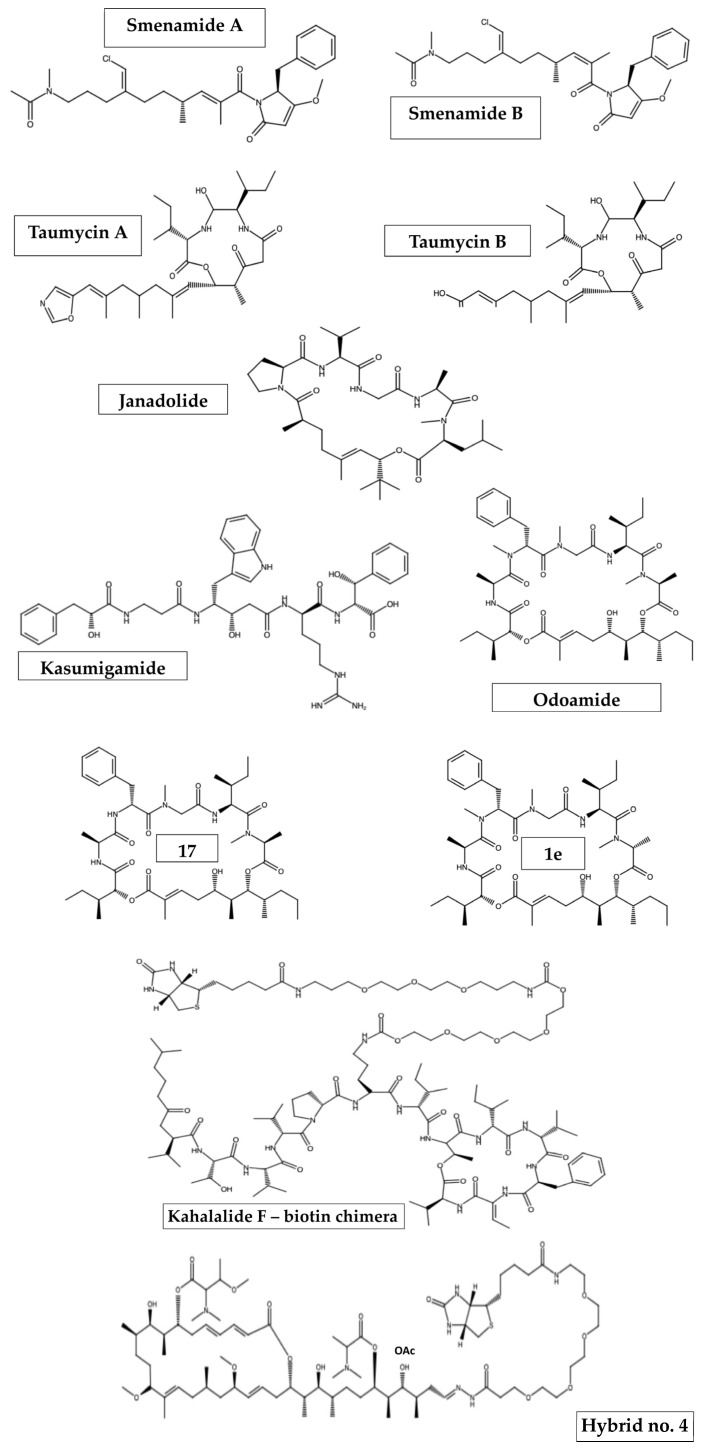
Representative structures of natural and synthetic marine-based chimeras.

Some marine organisms were found to have hybrid compounds per se. A suitable example of this is a paper by Teta et al. [109], which showed that marine sponges from the family Thorectida, in particular *Smenospongia aurea*, are rich in chlorinated hybrid peptide/polyketide compounds called smenamides A and B (Figure 2). Their unusual properties and biological behavior have been attributed to their structure, as they possess a western *N*-methylacetamide terminus, the dolapyrrolidone eastern terminus and the chlorovinyl functional group common to some cyanobacterial metabolites [110]. Consequently, both compounds were characterized by their cytotoxic activity. In fact, smenamide A and B show antiproliferative activity against lung cancer Calu-1 cells at nanomolar concentrations, with IC_50_ of 48 and 49 nM, respectively, via a clear pro-apoptotic mechanism [109,111]. Furthermore, smenamide A, together with its structural analogs, was presented to induce a dose-dependent effect against human multiple myeloma (MM) cell lines (i.e., SKM-M1 and RPMI-8226) [111].

*Okeania* sp., a marine cyanobacterium, also has natural hybrid compounds. One of these is janadolide (Figure 2), a new cyclic depsipeptide that is a peptide–polyketide hybrid with a *tert*-butyl group [112]. Janadolide showed potent antitrypanosomal activity against *Trypanosoma brucei*, *Trypanosoma rhodesiense* and *Trypanosoma cruzi* parasites, with an IC_50_ of 47 nM, 91.6 μM and 69.3 μM, respectively [112]. However, both janadolide and a number of its analogs were inactive against *Leishmania donovani* [113]. Moreover, even at 10 μM, janadolide had no cytotoxic effects against human cells, such as MRC-5, HL60 or HeLa cells [112], thus indicating its selective activity between harmful and harmless cells.

In addition to janadolide isolated from the Okinawa marine cyanobacterium *Okeania* sp., other peptide–polyketide hybrids have also been found. These include depsipeptides of the aurilide class, such as odoamide (Figure 2). This compound consists of three substructures: a polyketide unit, a peptide segment (Ala-*D*-MePhe-Sar-Ile-MeAla) and isoleucic acid [114]. Odoamide has been shown to be highly cytotoxic against the human cervical cancer cell line HeLa S3 [115] and the human lung cancer cell line A549, with IC_50_ values of 26.3 nM and 4.2 nM, respectively [116]. However, this has also been shown for some of the macrocyclic odoamide analogs prepared by Kaneda and colleagues [116], such as the *D*-MeAla6 epimer (compound **1e**; Figure 2) which showed slightly stronger bioactivity against A549 cells (IC_50_ = 1.9 nM) as compared to that of odoamide or peptide 17 (Figure 2) with a *D*-Phe3 substituent, which also exhibited high cytotoxicity (IC_50_ = 5.4 nM) [114,116].

In 2000, Ishida and Murakami determined the structure of another hybrid peptide–polyketide, which was again isolated from the cyanobacterium *Microcystis aureginosa* (NIES-87) [117]. However, further analyses showed that it was possibly produced by ‘*Entotheonella*’ sp., a bacterial symbiont phylotype identified in the Japanese marine sponge Discodermia calyx [118]. Kasumigamide (Figure 2), a linear tetrapeptide with an α-hydroxy acid located at the *N*-terminus, was thought to be responsible for the defensive activity of the cyanobacterium against the green algae *Chlamydomas neglecta* (NIES-439). However, no further studies are available that could provide additional information on the biological activities of kasumigamide.

It is noteworthy that, in the group of compounds displaying hybrid peptide–polyketide scaffolds, other intriguing marine-derived structures might be included, such as stereocalpin A and taumycins A and B (Figure 2) [119,120].

**Figure 3:**
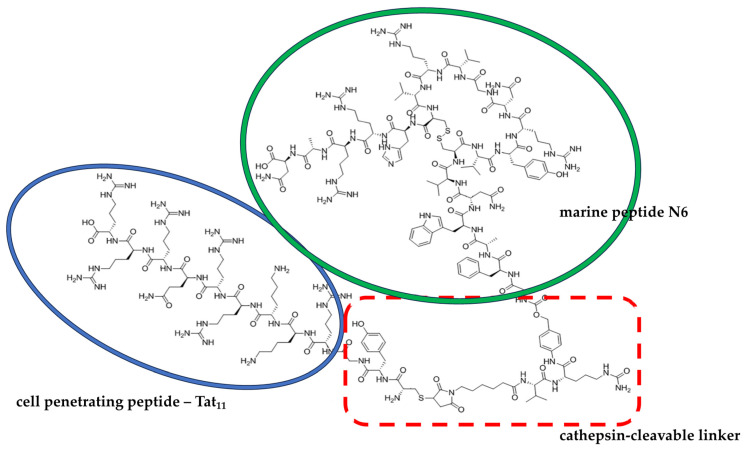
Molecular structure of compound **6**, which combines the marine peptide N6 and the cell-penetrating peptide Tat11, and was designed by Li et al. [123].

Recently, a peptide called N6 (Gly-Phe-Ala-Trp-Asn-Val-Cys-Val-Tyr-Arg-Asn-Gly-Val-Arg-Val-Cys-His-Arg-Arg-Ala-Asn), being an arenicin-3 derivative isolated from the lugworm, gained much interest owing to its potent antimicrobial activity [121,122]. In a study by Li and colleagues [123], a series of chimeras were developed that combine the N6 marine peptide and a cell-penetrating peptide, Tat_11_, isolated from the human immunodeficiency virus type 1 (HIV-1) via the cathepsin-cleavable linker MC-VC-PABC (maleimidocaproyl-L-valine-L-citrulline-*p*-aminobenzylcarbonyl), and both the in vitro and in vivo antimicrobial activities were evaluated against *Salmonellea typhimurium*. While one of the leading hybrids—compound no. **6** (Figure 3), with an MIC > 27.2 μM—showed no antibacterial activity, its antimicrobial activity was improved when the bacteria were pretreated with cathepsin B, reaching an MIC of 1.7 μM. Also, in a peritonitis mouse model, intraperitoneal (i.p.) treatment with compound **6** of mice injected with *S. typhimurium* improved the survival rate of mice (66.7%) and inhibited the growth of *S. typhimurium*.

This group also conjugated the aforementioned marine peptide N6 (at the *N*-terminus, *C*-terminus or at cysteine 7 or 16) with a series of linear glycol polyethylenes (PEG; *n* = 2, 6, 12 and 24) to improve its stability against trypsin. In this context, seven new PEGylated N6 analogs were designed with the most potent antimicrobial compound, N6-COOH-miniPEG (*n* = 2; Gly-Phe-Ala-Trp-Asn-Val-Cys-Val-Tyr-Arg-Asn-Gly-Val-Arg-Val-Cys-His-Arg-Arg-Ala-Asn–miniPEG). Indeed, that conjugate was active against both gram-negative and gram-positive bacterial strains and demonstrated higher antibacterial activity against gram-negative strains (with MICs of 1.53 for *Escherichia coli* and 24.42 μM for *Pseudomonas aeruginosa*) than against gram-positive bacteria (MICs ≥ 24.42 μM for *Staphylococcus aureus* and *Staphylococcus hyicus*). Remarkably, the compound also showed negligible hemolysis and improved proteolytic stability as compared to that of N6, as confirmed by an inhibition zone assay. In contrast, the PEGylation of N6 at the *N*-terminus showed either low or no antimicrobial activity [122].

A hybridization strategy was also utilized not only to improve the properties and activity of the mature compound but also to identify its molecular target. This applies to kahalalide F, isolated from the marine mollusk *Elysia rufescens* [124], and aplyronine A from the Japanese sea hare *Aplysia kurodai* [125]. Kahalalide F, a potent anticancer molecule, was conjugated with a biotinylated linker to give compound “kahalalide-biotin” (Figure 2), for which human ribosomal protein S25 was found to be responsible for its potent anticancer properties in a dose-dependent manner [126]. Meanwhile, condensation of aplyronine A with a PEG-linked biotin hydrazide yielded compound **4** (Figure 2), a less potent derivative compared to that of aplyronine A (IC_50_ = 0.096 nM vs. 0.010 nM, respectively, against the HeLa S3 cell line) and with similar depolymerizing activity to both fiver actin and the parent compound [125].

It is noteworthy that none of the presented compounds have been described in terms of their off-target side effects. This confirms that the current research on these new hybrids is still at an early stage and is aimed at determining their biological activities and comparing them with the parent substances that constitute the chimera-building pharmacophores.

## 5. Conclusions

Compounds isolated from marine plants, sponges and microorganisms have repeatedly demonstrated their high efficacy and their variety of important properties, including antibacterial, antidiabetic and anticancer functions. Such a wealth of pharmacologically active structures could be of great importance in a world of expanding bacterial resistance and constant battles against ever more life-threatening diseases. Furthermore, these molecules can serve as excellent building blocks for chimeric compounds proposed for the treatment of various complex diseases, especially when considering that current drug discovery strategies predominantly emphasize single molecule-based treatments. Importantly, the sometimes high side effect profile reported for most marine compounds at therapeutic doses can be minimized by the chemical binding of the compound to another biologically active compound, as is the case in hybrids. Hence, the toxicity of the parent compounds does not always translate into the toxicity of a hybrid composed of such compounds. However, given the unique mechanism of action of such molecules and the fact that hybrids themselves are known to be imperfectly identical in nature to their individual components, a thorough evaluation of the activity of the hybrids, including pharmacokinetics and toxicological profiles, should also be undertaken.

Unfortunately, there is still little to no information on the off-target activity of such structures. On the other hand, it seems unreasonable to specify such activity here for structures unrelated to marine peptides. Nevertheless, we should keep them in mind, even if they may prove beneficial in some cases.

This review has some limitations, as only a few marine sources were characterized and, hence, the full potential of marine life could not be presented. In addition, this review focuses on marine peptides, while many other different chemicals have been discovered, including phenolic compounds, fatty acids, polyethers, carbohydrates, etc. Nevertheless, this review clearly shows that marine-derived compounds can serve as an excellent source of inspiration and as a starting point for drug development, and in this context, should attract much attention as potent, active pharmacophores for very complex molecules of a hybrid nature. Obviously, it should be kept in mind that, as the marine environment is extremely diverse, an unknown number of additional marine peptides remain to be identified and tested.

## Figures and Tables

**Table 2:** A one-letter code of amino acid sequence of the chimeric peptide SNX-202, with highlighted constituent pharmacophores and indications of their origins.

Compound	Amio Acid Sequence
SNX-111	CKGKGAKCSRLMYDCCTGSCRSGKC
SNX-183	CKLKGQSCRKTSYDCCSGSCGRRRGKC
SNX-202 *	CKLKGQSC SRLMY DCCSGSCGRRRG

* SNX-202 is a synthetic hybrid peptide containing amino acids 1 to 8 and 13 to 25 of SNX-183 and residues 9 to 12 of SNX-111. The colors represent amino acid fragments that correspond to both the naturally occurring SNX-111 peptide (red) and SNX-183 (green).

## References

[1] Kleczkowska P. (2022). Chimeric structures in mental illnesses—“Magic” Molecules Specified for Complex Disorders. Int. J. Mol. Sci..

[2] Abdolmaleki A., Ghasemi J.B. (2017). Dual-acting of hybrid compounds—A New Dawn in the Discovery of Multi-target Drugs: Lead Generation Approaches. Curr. Top. Med. Chem..

[3] Müller-Schiffmann A., Sticht H., Korth C. (2012). Hybrid compounds: From simple combinations to nanomachines. BioDrugs.

[4] Korth C., Klingenstein R., Müller-Schiffmann A. (2013). Hybrid molecules synergistically acting against protein aggregation diseases. Curr. Top. Med. Chem..

[5] Muchowska A., Redkiewicz P., Różycki K., Matalińska J., Lipiński P.F.J., Czuwara J., Kosson P. (2020). The analgesic hybrid of dermorphin/substance P and analog of enkephalin improve wound healing in streptozotocin-induced diabetic rats. Wound Repair Regen..

[6] Foran S.E., Carr D.B., Lipkowski A.W., Maszczynska I., Marchand J.E., Misicka A., Beinborn M., Kopin A.S., Kream R.M. (2000). Inhibition of morphine tolerance development by a substance P-opioid peptide chimera. J. Pharmacol. Exp. Ther..

[7] Mollica A., Costante R., Stefanucci A., Pinnen F., Luisi G., Pieretti S., Borsodi A., Bojnik E., Benyhe S. (2013). Hybrid peptides endomorphin-2/DAMGO: Design, synthesis and biological evaluation. Eur. J. Med. Chem..

[8] Wagner H., Efferth T. (2017). Introduction: Novel hybrid combinations containing synthetic or antibiotic drugs with plant-derived phenolic or terpenoid compounds. Phytomedicine.

[9] Xu W., Popovich D.G. (2023). Bioactive hybrid compounds from Myrtaceae: Chemical classification and biological activities. Stud. Natur. Prod. Chem..

[10] Pratt J.H. (1954). A reappraisal of researches leading to the discovery of insulin. J. Hist. Med..

[11] Wodlej C., Riedl S., Rinner B., Leber R., Drechsler C., Voelker D.R., Choi J.-Y., Lohner K., Zweytick D. (2019). Interaction of two antitumor peptides with membrane lipids–Influence of phosphatidylserine and cholesterol on specificity for melanoma cells. PLoS ONE.

[12] Anjum K., Abbas S.Q., Akhter N., Shagufta B.I., Shah S.A.A., Hassan S.S.U. (2017). Emerging biopharmaceuticals from bioactive peptides derived from marine organisms. Chem. Biol. Drug Des..

[13] Mayer A.M.S., Rodríguez A.D., Taglialatela-Scafati O., Fusetani N. (2013). Marine pharmacology in 2009–2011: Marine compounds with antibacterial, antidiabetic, antifungal, anti-inflammatory, antiprotozoal, antituberculosis, and antiviral activities; affecting the immune and nervous systems, and other miscellaneous mechanisms of action. Mar. Drugs.

[14] Sheih I.C., Wu T.K., Fang T.J. (2009). Antioxidant properties of a new antioxidative peptide from algae protein waste hydrolysate in different oxidation systems. Bioresour. Technol..

[15] Admassu H., Gasmalla M.A.A., Yang R., Zhao W. (2018). Bioactive peptides derived from seaweed protein and their health benefits: Antihypertensive, antioxidant, and antidiabetic properties. J. Food Sci..

[16] Negi B., Kumar D., Rawat D.S. (2017). Marine peptides as anticancer agents: A remedy to mankind by nature. Curr. Protein Pept. Sci..

[17] Kleczkowska P., Kosson P., Ballet S., Van den Eynde I., Tsuda Y., Tourwé D., Lipkowski A.W. (2010). PK20, a new opioid-neurotensin hybrid peptide that exhibits central and peripheral antinociceptive effects. Mol. Pain..

[18] Silbert B.S., Lipkowski A.W., Cepeda M.S., Szyfelbein S.K., Osgood P.F., Carr D.B. (1991). Analgesic activity of novel bivalent opioid peptide compared to morphine via different routes administration. Agents Action.

[19] Kleczkowska P., Hermans E., Kosson P., Kowalczyk A., Lesniak A., Pawlik K., Bojnik E., Benyhe S., Nowicka B., Bujalska-Zadrozny M. (2016). Antinociceptive effect induced by a combination of opioid and neurotensin moieties vs. their hybrid peptide [Ile9]PK20 in an acute pain treatment in rodents. Brain Res..

[20] Klingenstein R., Lober S., Kujala P., Godsave S., Leliveld S.R., Gmeiner P., Peters P.J., Korth C. (2006). Tricyclic antidepressants, quinacrine and a novel, synthetic chimera thereof clear prions by destabilizing detergent-resistant membrane compartments. J. Neurochem..

[21] Kleczkowska P., Kawalec M., Bujalska-Zadrozny M., Filip M., Zablocka B., Lipkowski A.W. (2015). Effects of the hybridization of opioid and neurotensin pharmacophores on cell survival in rat organotypic hippocampal slice cultures. Neurotox. Res..

[22] Bądzyńska B., Lipkowski A.W., Sadowski J. (2016). An antihypertensive opioid: Biphalin, a synthetic non-addictive enkephalin analog decreases blood pressure in spontaneously hypertensive rats. Pharmacol. Rep..

[23] Sang Z., Li Y., Qiang X., Xiao G., Liu Q., Tan Z., Deng Y. (2015). Multifunctional scutellarin–rivastigmine hybrids with cholinergic, antioxidant, biometal chelating and neuroprotective properties for the treatment of Alzheimer’s disease. Bioorg. Med. Chem..

[24] Diao L., Meibohm B. (2013). Pharmacokinetics and pharmacokinetic-pharmacodynamic correlations of therapeutic peptides. Clin. Pharmacokinet..

[25] Wang L., Wang N., Zhang W., Cheng X., Yan Z., Shao G., Wang X., Wang R., Fu C. (2022). Therapeutic peptides: Current applications and future directions. Signal Transduct. Target Ther..

[26] Wetzler M., Hamilton P. (2018). Peptides as therapeutics. Peptide Applications in Biomedicine, Biotechnology and Bioengineering.

[27] Craik D.J., Fairlie D.P., Liras S., Price D. (2013). The future of peptide-based drugs. Chem. Biol. Drug Des..

[28] Barman P., Joshi S., Sharma S., Preet S., Sharma S., Saini A. (2023). Strategic approaches to improvise peptide drugs as next generation therapeutics. Int. J. Pept. Res. Ther..

[29] Shinnar A.E., Butler K.L., Park H.J. (2003). Cathelicidin family of antimicrobial peptides: Proteolytic processing and protease resistance. Bioorg. Chem..

[30] Lee Y., Phat C., Hong S.C. (2017). Structural diversity of marine cyclic peptides and their molecular mechanisms for anticancer, antibacterial, antifungal, and other clinical applications. Peptides.

[31] Schultz A.W., Oh D.C., Carney J.R., Williamson R.T., Udwary D.W., Jensen P.R., Gould S.J., Fenical W., Moore B.S. (2008). Biosynthesis and structures of cyclomarins and cyclomarazines, prenylated cyclic peptides of marine actinobacterial origin. J. Am. Chem. Soc..

[32] Pettit G.R., Cichacz Z., Barkoczy J., Dorsaz A.C., Herald D.L., Williams M.D., Doubek D.L., Schmidt J.M., Tackett L.P., Brune D.C. (1993). Isolation and structure of the marine sponge cell growth inhibitory cyclic peptide phakellistatin 1. J. Nat. Prod..

[33] Destoumieux D., Munoz M., Bulet P., Bachere E. (2000). Penaeidins, a family of antimicrobial peptides from penaeid shrimp. Cell. Mol. Life Sci..

[34] Wu R., Patocka J., Nepovimova E., Oleksak P., Valis M., Wu W., Kuca K. (2021). Marine invertebrate peptides: Antimicrobial peptides. Front. Microbiol..

[35] Pan W., Liu X., Ge F., Han J., Zheng T. (2004). Perinerin, a novel antimicrobial peptide purified from the clamworm Perinereis aibuhitensis grube and its partial characterization. J. Biochem..

[36] Chaturvedi P., Bhat R.A.H., Pande A. (2020). Antimicrobial peptides of fish: Innocuous alternatives to antibiotics. Rev. Aquac..

[37] Kapil S., Sharma V. (2021). D-Amino acids in antimicrobial peptides: A potential approach to treat and combat antimicrobial resistance. Can. J. Microbiol..

[38] Phyo Y.Z., Ribeiro J., Fernandes C., Kijjoa A., Pinto M.M.M. (2018). Marine natural peptides: Determination of absolute configuration using liquid chromatography methods and evaluation of bioactivities. Molecules.

[39] Aillaud I., Kaniyappan S., Chandupatla R.R., Ramirez L.M., Alkhashrom S., Eichler J., Horn A.H.C., Zweckstetter M., Mandelkow E., Sticht H. (2022). A novel D-amino acid peptide with therapeutic potential (ISAD1) inhibits aggregation of neurotoxic disease-relevant mutant Tau and prevents Tau toxicity in vitro. Alzheimers Res. Ther..

[40] Lu J., Xu H., Xia J., Ma J., Xu J., Li Y., Feng J. (2020). D- and unnatural amino acid substituted antimicrobial peptides with improved proteolytic resistance and their proteolytic degradation characteristics. Front. Microbiol..

[41] Pavlicevic M., Maestri E., Marmiroli M. (2020). Marine bioactive peptides-an overview of generation, structure and application with a focus on food sources. Mar. Drugs.

[42] Giordano D. (2020). Bioactive molecules from extreme environments. Mar. Drugs.

[43] Alonzo D.A., Schmeing T.M. (2020). Biosynthesis of depsipeptides, or Depsi: The peptides with varied generations. Protein Sci..

[44] Zeng M., Tao J., Xu S., Bai X., Zhang H. (2023). Marine organisms as a prolific source of bioactive depsipeptides. Mar. Drugs.

[45] Nič M., Jirát J., Košata B., Jenkins A., McNaught A. (2009). IUPAC Compendium of Chemical Terminology.

[46] Ariyoshi Y. (1993). Angiotensin-converting enzyme inhibitors derived from food proteins. Trends Food Sci. Technol..

[47] Ukeda H., Matsuda H., Kuroda H., Osajima K., Matsufuji H., Osajima Y. (1991). Preparation and separation of angiotensin I converting enzyme inhibitory peptides. Nippon Nogeikagaku Kaishi.

[48] Ranathunga S., Rajapakse N., Kim S.-K. (2006). Purification and characterization of antioxidative peptide derived from muscle of conger eel (*Conger myriaster*). Eur. Food Res. Technol..

[49] Kohama Y., Matsumoto S., Oka H., Teramoto T., Okabe M., Mimura T. (1988). Isolation of angiotensin-converting enzyme-inhibitor from tuna muscle. Biochem. Biophys. Res. Commun..

[50] Guo X., Liu J., Wang C., Wen Z., Zheng B. (2024). The antioxidant mechanism of peptides extracted from tuna protein revealed using a molecular docking simulation. Antioxidants.

[51] Kim S.Y., Je J.Y., Kim S.K. (2007). Purification and characterization of antioxidant peptide from hoki (*Johnius belengerii*) frame protein by gastrointestinal digestion. J. Nutr. Biochem..

[52] Hsu K.-C., Li-Chan E.C., Jao C.-L. (2011). Antiproliferative activity of peptides prepared from enzymatic hydrolysates of tuna dark muscle on human breast cancer cell line MCF-7. Food Chem..

[53] Zhao X., Cai B., Chen H., Wan P., Chen D., Ye Z., Duan A., Chen X., Sun H., Pan J. (2023). Tuna trimmings (*Thunnas albacares*) hydrolysate alleviates immune stress and intestinal mucosal injury during chemotherapy on mice and identification of potentially active peptides. Curr. Res. Food Sci..

[54] Lee S.H., Qian Z.J., Kim S.K. (2010). A novel angiotensin I converting enzyme inhibitory peptide from tuna frame protein hydrolysate and its antihypertensive effect in spontaneously hypertensive rats. Food Chem..

[55] Suo S.-K., Zheng S.-L., Chi C.-F., Luo H.-Y., Wang B. (2022). Novel angiotensin-converting enzyme inhibitory peptides from tuna byproducts-milts: Preparation, characterization, molecular docking study, and antioxidant function on H_2_O_2_-damaged human umbilical vein endothelial cells. Front. Nutr..

[56] Wu H., He H.L., Chen X.L., Sun C.Y., Zhang Y.Z., Zhou B.C. (2008). Purification and identification of novel angiotensin-I-converting enzyme inhibitory peptides from shark meat hydrolysate. Process Biochem..

[57] Cho J., Kim Y. (2002). Sharks: A potential source of antiangiogenic factors and tumor treatments. Mar. Biotechnol..

[58] Kern B.E., Balcom J.H., Antoniu B.A., Warshaw A.L., Fernández-del Castillo C. (2003). Troponin I peptide (Glu94-Leu123), a cartilage-derived angiogenesis inhibitor: In vitro and in vivo effects on human endothelial cells and on pancreatic cancer. J. Gastrointest. Surg..

[59] Huang F.J., Lv Z.B., Li Q., Wei L.J., Zhang L., Wu W.T. (2005). Study on hepatoprotective effect of peptide S-8300 from shark liver. World J. Gastroenterol..

[60] Huang F., Wu W. (2005). Antidiabetic effect of a new peptide from *Squalus mitsukurii* liver (S-8300) in alloxan-diabetes. Clin. Exp. Pharmacol. Physiol..

[61] Huang T.C., Lee J.F., Chen J.Y. (2011). Pardaxin, an antimicrobial peptide, triggers caspase-dependent and ROS-mediated apoptosis in HT-1080 cells. Mar. Drugs.

[62] Hsu J.C., Lin L.C., Tzen J.T.C., Chen J.Y. (2011). Pardaxin-induced apoptosis enhances antitumor activity in HeLa cells. Peptides.

[63] Mulero I., Noga E.J., Meseguer J., Garcia-Ayala A., Mulero V. (2008). The antimicrobial peptides piscidins are stored in the granules of professional phagocytic granulocytes of fish and are delivered to the bacteria-containing phagosome upon phagocytosis. Dev. Comp. Immunol..

[64] Asensio-Calavia P., González-Acosta S., Otazo-Pérez A., López M.R., Morales-delaNuez A., Pérez de la Lastra J.M. (2023). Teleost piscidins-in silico perspective of natural peptide antibiotics from marine sources. Antibiotics.

[65] Wang Y.D., Kung C.W., Chen J.Y. (2010). Antiviral activity by fish antimicrobial peptides of epinecidin-1 and hepcidin 1–5 against nervous necrosis virus in medaka. Peptides.

[66] Pan C.Y., Chen J.Y., Lin T.L., Lin C.H. (2009). In vitro activities of three synthetic peptides derived from epinecidin-1 and an anti-lipopolysaccharide factor against *Propionibacterium acnes*, *Candida albicans*, and *Trichomonas vaginalis*. Peptides.

[67] Niu S.F., Jin Y., Xu X., Qiao Y., Wu Y., Mao Y., Su Y.Q., Wang J. (2013). Characterization of a novel piscidin-like antimicrobial peptide from *Pseudosciaena crocea* and its immune response to *Cryptocaryon irritans*. Fish Shellfish Immunol..

[68] Pereiro P., Figueras A., Novoa B. (2012). A novel hepcidin-like in turbot (*Scophthalmus maximus* L.) highly expressed after pathogen challenge but not after iron overload. Fish Shellfish Immunol..

[69] Pan C.Y., Lee S.C., Rajanbabu V., Lin C.H., Chen J.Y. (2012). Insights into the antibacterial and immunomodulatory functions of tilapia hepcidin (TH)2–3 against Vibrio vulnificus infection in mice. Dev. Comp. Immunol..

[70] Chen J.Y., Lin W.J., Lin T.L. (2009). A fish antimicrobial peptide, tilapia hepcidin TH2–3, shows potent antitumor activity against human fibrosarcoma cells. Peptides.

[71] Hsu J.C., Lin L.C., Tzen J.T., Chen J.Y. (2011). Characteristics of the antitumor activities in tumor cells and modulation of the inflammatory response in RAW264.7 cells of a novel antimicrobial peptide, chrysophsin-1, from the red sea bream (*Chrysophrys major*). Peptides.

[72] Lin W.J., Chien Y.L., Pan C.Y., Lin T.L., Chen J.Y., Chiu S.J., Hui C.F. (2009). Epinecidin-1, an antimicrobial peptide from fish (Epinephelus coioides) which has an antitumor effect like lytic peptides in human fibrosarcoma cells. Peptides.

[73] Hilchie A.L., Doucette C.D., Pinto D.M., Patrzykat A., Douglas S., Hoskin D.W. (2011). Pleurocidin-family cationic antimicrobial peptides are cytolytic for breast carcinoma cells and prevent growth of tumor xenografts. Breast Cancer Res..

[74] Rigano F., Arena P., Mangraviti D., Donnarumma D., Dugo P., Donato P., Mondello L., Micalizzi G. (2021). Identification of high-value generating molecules from the wastes of tuna fishery industry by liquid chromatography and gas chromatography hyphenated techniques with automated sample preparation. J. Sep. Sci..

[75] Qian Z.-J., Je J.-Y., Kim S.-K. (2007). Antihypertensive effect of angiotensin I converting enzyme inhibitory peptide from hydrolysates of bigeye tuna dark muscle, *Thunnus obesus*. J. Agric. Food Chem..

[76] Wang X., Yu H., Xing R., Chen X., Li R., Li K., Liu S., Li P. (2018). Purification and identification of antioxidative peptides from mackerel (pneumatophorus japonicus) protein. RSC Adv..

[77] Pan C.-Y., Lin C.-N., Chiou M.-T., Yu C.Y., Chen J.-Y., Chien C.-H. (2015). The antimicrobial peptide pardaxin exerts potent anti-tumor activity against canine perianal gland adenoma. Oncotarget.

[78] Abu-Raya S., Bloch-Shilderman E., Lelkes P.I., Trembovler V., Shohami E., Gutman Y., Lazarovici P. (1999). Characterization of pardaxin-induced dopamine release from pheochromocytoma cells: Role of calcium and eicosanoids. J. Pharmacol. Exp. Ther..

[79] Lazarovici P. (2002). The structure and function of pardaxin. J. Toxicol. Toxin Rev..

[80] Jung W.K., Mendis E., Je J.Y., Park P.J., Son B.W., Kim H.C., Choi Y.K., Kim S.K. (2005). Angiotensin I-converting enzyme inhibitory peptide from yellowfin sole *(Limanda aspera)* frame protein and its antihypertensive effect in spontaneously hypertensive rats. Food Chem..

[81] Bhusal A., Nam Y., Seo D., Rahman M.H., Hwang E.M., Kim S.C., Lee W.H., Suk K. (2022). Cathelicidin-related antimicrobial peptide promotes neuroinflammation through astrocyte-microglia communication in experimental autoimmune encephalomyelitis. Glia.

[82] Basanez G., Shinnar A.E., Zimmerberg J. (2002). Interaction of hagfish cathelicidin antimicrobial peptides with model lipid membranes. FEBS Lett..

[83] Ngo D.H., Ryu B., Vo T.S., Himaya S.W.A., Wijesekara I., Kim S.K. (2011). Free radical scavenging and angiotensin-I converting enzyme inhibitory peptides from Pacific cod (*Gadus macrocephalus*) skin gelatin. Int. J. Biol. Macromol..

[84] Wang D., Huang X., Marnila P., Hiidenhovi J., Valimaa A.L., Granato D., Makinen S. (2004). Baltic herring hydrolysates: Identification of peptides, in silico DPP-4 prediction, and their effects on an in vivo mice model of obesity. Food Res. Int..

[85] Ono S., Hosokawa M., Miyashita K., Takahashi K. (2003). Isolation of peptides with angiotensin I-converting enzyme inhibitory effect derived from hydrolysate of upstream chum salmon muscle. J. Food Sci..

[86] Li-Chan E.C.Y., Hunag S.-L., Jao C.-L., Ho K.-P., Hsu K.-C. (2012). Peptides derived from atlantic salmon skin gelatin as dipeptidyl-peptidase IV inhibitors. J. Agric. Food Chem..

[87] Matsufuji H., Matsui T., Seki E., Osajima K., Nakashima H., Osajima K. (1994). Angiotensin Iconverting enzyme inhibitory peptides in an alkaline protease hydrolyzate derived from sardine muscle. Biosci. Biotechnol. Biochem..

[88] Cruz L.J., de Santoz V., Zafaralla G.C., Ramilo C.A., Zeikus R., Gray W.R., Olivera B.M. (1987). Invertebrate vasopressin/oxytocin homologs. Characterization of peptides from *Conus geographus* and *Conus straitus* venoms. J. Biol. Chem..

[89] Cottrell G.A., Twarog B.M. (1972). Proceedings: Active factors in the venom duct of Conus californicus. Br. J. Pharmacol..

[90] Green B.R., Olivera B.M. (2016). Venom peptides from cone snails: Pharmacological Probes for Voltage-Gated Sodium Channels. Curr. Top Membr..

[91] Safavi-Hemami H., Brogan S.E., Olivera B.M. (2019). Pain therapeutics from cone snail venoms: From Ziconotide to novel non-opioid pathways. J. Proteom..

[92] Rauck R.L., Wallac M.S., Leong M.S., Minehart M., Webster L.R., Charapata S.G., Abraham J.E., Buffington D.E., Ellis D., Kartzinel R. (2006). A randomized, double-blind, placebo-controlled study of intrathecal ziconotide in adults with severe chronic pain. J. Pain Symptom Manag..

[93] Frank M., Hiu L.W., Begley T.P. (2010). Natural Peptide Toxins. Comprehensive Natural Products.

[94] Ohizumi Y., Nakamura H., Kobayashi J. (1986). Presynaptic inhibitory effect of geographutoxin II, a new peptide toxin from *Conus geographus* venom, in the guinea-pig vas deferens. Eur. J. Pharmacol..

[95] Ohizumi Y., Minoshima S., Takahashi M., Kajiwara A., Nakamura H., Kobayashi J. (1986). Geographutoxin II, a novel peptide inhibitor of Na channels of skeletal muscles and autonomic nerves. J. Pharmacol. Exp. Ther..

[96] Ohizumi Y., Nakamura H., Kobayashi J., Catterall W.A. (1986). Specific inhibition of [3H]saxitoxin binding to skeletal muscle sodium channels by geographutoxin II, a polypeptide channel blocker. J. Biol. Chem..

[97] Moczydlowski E., Olivera B.M., Gray W.R., Strichartz G.A. (1986). Discrimination of muscle and neuronal Na-channel subtypes by binding competition between [3H]saxitoxin and p-conotoxins. Proc. Natl. Acad. Sci. USA.

[98] Layer R.T., McIntosh J.M. (2006). Conotoxins: Therapeutic Potential and Application. Mar. Drugs.

[99] Malmberg A.B., Gilbert H., McCabe R.T., Basbaum A.I. (2003). Powerful antinociceptive effects of the cone snail venom-derived subtype-selective NMDA receptor antagonists conantokins G and T. Pain.

[100] Sánchez A., Vázquez A. (2017). Bioactive peptides: A review. Food Qual. Saf..

[101] Fan X., Bai L., Mao X., Zhang X. (2017). Novel peptides with anti-proliferation activity from the *Porphyra haitanesis* hydrolysate. Process Biochem..

[102] Suetsuna K., Chen J.-R. (2001). Identification of antihypertensive peptides from peptic digest of two microalgae, *Chlorella vulgaris* and Spirulina platensis. Mar. Biotechnol..

[103] Chen M.-F., Zhang Y.Y., Di He M., Li C.Y., Zhou C.X., Hong P.Z., Qian Z.-J. (2019). Antioxidant peptide purified from enzymatic hydrolysates of isochrysis zhanjiangensis and its protective effect against ethanol induced oxidative stress of HepG2 Cells. Biotechnol. Bioprocess Eng..

[104] Ramachandran J. (1994). Structure, function and therapeutic potential of omega conopeptides: Novel blockers of neuronal calcium channels. Proc. Indian Acad. Sci (Chem. Sci.).

[105] Nadasdi L., Yamashiro D., Chung D., Tarczy-Hornoch K., Adriaenssens P., Ramachandran J. (1995). Structure-activity analysis of a conus peptide blocker of n-type neuronal calcium channels. Biochemistry.

[106] Ekberg J., Craik D.J., Adams D.J. (2008). Conotoxin modulation of voltage-gated sodium channels. Int. J. Biochem. Cell Biol..

[107] Ohashi N., Uta D., Ohashi M., Hoshino R., Baba H. (2024). Omega-conotoxin MVIIA reduces neuropathic pain after spinal cord injury by inhibiting N-type voltage-dependent calcium channels on spinal dorsal horn. Front. Neurosci..

[108] Tombaccini D., Adeyemo O.M., Pollard H.B., Feuerstein G. (1990). Monoclonal antibodies against the presynaptic calcium channel antagonist ω-conotoxin GVI A from cone snail poison. FEBS Lett..

[109] Teta R., Irollo E., Della Sala G., Pirozzi G., Mangoni A., Costantino V. (2013). Smenamides A and B, chlorinated peptide/polyketide hybrids containing a dolapyrrolidinone unit from the Caribbean sponge *Smenospongia aurea*. Evaluation of their role as leads in antitumor drug research. Mar. Drugs.

[110] Edwards D.J., Marquez B.L., Nogle L.M., McPhail K., Goeger D.E., Roberts M.A., Gerwick W.H. (2004). Structure and biosynthesis of the Jamaicamides, New Mixed Polyketide-Peptide Neurotoxins from the Marine Cyanobacterium *Lyngbya majuscule*. Chem. Biol..

[111] Caso A., Laurenzana I., Lamorte D., Trino S., Esposito G., Piccialli V., Costantino V. (2018). Smenamide A analogues. Synthesis and biological activity on multiple myeloma cells. Mar. Drugs.

[112] Ogawa H., Iwasaki A., Sumimoto S., Kanamori Y., Ohno O., Iwatsuki M., Ishiyama A., Hokari R., Otoguro K., Ōmura S. (2016). Janadolide, a cyclic polyketide-peptide hybrid possessing a tert-butyl group from an *Okeania sp.* marine cyanobacterium. J. Nat. Prod..

[113] Chung J.H., Tang A.H., Geraghty K., Corcillus L., Kaiser M., Payne R.J. (2020). Total synthesis and antitripanosomal activity of janadolide and simplified analogues. Org. Lett..

[114] https://repository.kulib.kyoto-u.ac.jp/dspace/bitstream/2433/232326/2/dykkk00092.pdf.

[115] Sueyoshi K., Kaneda M., Sumimoto S., Oishi S., Fujii N., Suenaga K., Teruya T. (2016). Odoamide, a cytotoxic cyclodepsipeptide from the marine cyanobacterium *Okeanina* sp.. Tetrahedron.

[116] Kaneda M., Kawaguchi S., Fujii N., Ohno H., Oishi S. (2018). Structure-activity relationship study on odoamide: Insights into the bioactivities of aurilide-family hybrid peptide-polyketides. ACS Med. Chem. Lett..

[117] Ishida K., Murakami M. (2000). Kasumigamide, an antialgal peptide from the cyanobacterium Microcystis aeruginosa. J. Org. Chem..

[118] Nakashima Y., Egami Y., Kimura M., Wakimoto T., Abe I. (2016). Metagenomic analysis of the sponge Discodermia reveals the production of the cyanobacterial natural product kasumigamide by ‘Entotheonella’. PLoS ONE.

[119] Seo C., Yim J.H., Lee H.K., Park S.M., Sohn J.H., Oh H. (2008). Stereocalpin A, a bioactive cyclic depsipeptide from the Antarctic lichen Stereocaulon alpinum. Tetrahedron Lett..

[120] Bishara A., Rudi A., Aknin M., Neumann D., Ben-Califa N., Kashman Y. (2008). Taumycin A and B, two bioactive lipodepsipeptides from the Madagascar sponge *Fascaplysinopsis* sp.. Org. Lett..

[121] Yang N., Liu X., Teng D., Li Z., Wang X., Mao R., Wang X., Hao Y., Wang J. (2017). Antibacterial and detoxifying activity of NZ17074 analogues with multi-layers of selective antimicrobial actions against *Escherichia coli* and *Salmonella enteritidis*. Sci. Rep..

[122] Li T., Yang N., Teng D., Mao R., Hao Y., Wang X., Wang J. (2022). C-terminal mini-PEGylation of a marine peptide N6 had potent antibacterial and anti-inflammatory properties against *Escherichia coli* and *Salmonella* strains in vitro and in vivo. BMC Microbiol..

[123] Li Z., Teng D., Mao R., Wang X., Hao Y., Wang X., Wang J. (2018). Improved antibacterial activity of the marine peptide N6 against intracellular salmonella typhimurium by conjugating with the cell-penetrating peptide Tat_11_via a cleavable linker. J. Med. Chem..

[124] Hamann M.T., Scheuer P.J., Kahalalide F. (1993). A bioactive depsipeptide from the sacoglossan mollusk *Elysia rufescens* and the green alga *Bryopsis* sp.. J. Am. Chem. Soc..

[125] Kita M., Hirayama Y., Sugiyama M., Kigoshi H. (2011). Development of highly cytotoxic and actin-depolymerizing biotin derivatives of Aplyronine A. Angew. Chemie.

[126] Piggott A.M., Karuso P. (2008). Rapid identification of a protein binding partner for the marine natural product kahalalide F by using reverse chemical proteomics. ChemBioChem.

